# Epidemiological profile of funguria in a university hospital in Oujda, Morocco

**DOI:** 10.18502/cmm.6.4.5328

**Published:** 2020-12

**Authors:** Adil Maleb, Aziza Hami, Somiya Lamrabat, Safae Rifai, Nawal Rahmani, Mohammed Bensalah, Elmostafa Benaissa, Yassine Ben Lahlou, Mohammed Frikh, Mostafa Elouennass

**Affiliations:** 1 Laboratory of Microbiology, Mohammed VI University Hospital/Faculty of Medicine and Pharmacy, Mohammed I University, Oujda, Morocco; 2 Laboratory of Parasitology-Mycology, Mohammed VI University Hospital/Faculty of Medicine and Pharmacy, Mohammed I University, Oujda, Morocco; 3 Department of Bacteriology, Mohammed V Military Teaching Hospital/Faculty of Medicine and Pharmacy, Mohammed V University, Rabat, Morocco

**Keywords:** Funguria, Infection, Urinary tract infection, Urine, Yeast

## Abstract

**Background and Purpose:**

The presence of yeasts in the urine is not synonymous with urinary tract infection since it can result in simple colonization or contamination. Regarding this, it is required to further clarify the epidemiological profile of funguria. Accordingly, the present study was conducted to establish the epidemiology of funguria in the Mohammed VI Teaching Hospital of Oujda, Morocco.

**Materials and Methods:**

This retrospective study was conducted on all urine samples sent for cytobacteriological examination to a microbiology laboratory over a period of 28 months (i.e., from March 2016 to June 2018). After the removal of duplicates, the urine samples were treated according to the recommendations of the medical microbiology standards.

**Results:**

A total of 15,165 urine samples were collected. Urinary colonization accounted for 4.94% (n=749) of cases. The infections of the urinary tract accounted for 5.35% (n=811) of cases. Microbial isolates (n=1,669) in colonization and urinary tract infections were dominated by bacteria (93.47%, n=1,560). Furthermore, the yeasts accounted for 6.53% (n=109) of the isolates. *Candida albicans* was isolated from 56.88% (n=62) of funguria cases. The risk factors for funguria in our series were essentially old age, admission to intensive care unit, and broad-spectrum antibiotic therapy.

**Conclusion:**

The current level of knowledge about the clinical situations leading to funguria with the improvement and popularization of efficient identification techniques for yeasts other than *C. albicans *should redress the epidemiology of funguria. This should allow the knowledgeable societies to establish the rules of interpreting the cytobacteriological examination of the urine in case of funguria, as for bacteriuria.

## Introduction

The presence of yeasts in the urine does not always mean that the urinary tract is infected since it could be colonization or a simple contamination. According to the literature, yeasts are found in 1-5% and 10-29% of the cytobacteriological examinations of the urine (CBEU) in the community and hospitals, respectively ( 1). There are few scientific studies and recommendations for the management of funguria, compared to those for bacteriuria. As a result, there is no consensus on funguria treatment ( 2- 4). However, it is generally agreed that *Candida albicans*is a dominant species in funguria ( 2- 5). 

With the multiplication of the causes of patient fragility, especially in a hospital context, and the emergence of yeasts from "non-*albicans Candida*" group, the precision of funguria epidemiology becomes a necessity. Regarding this, the objective of this study was to establish the epidemiology of funguria in the Mohammed VI University Teaching Hospital of Oujda, Morocco. 

## Materials and Methods

This retrospective study was conducted on all urine samples sent for cytobacteriological examination to the Microbiology Laboratory of the Mohammed VI University Teaching Hospital of Oujda for CBEU over a period of 28 months (i.e., from March 2016 to June 2018). After the removal of the duplicates, data related to patient demographics, underlying diseases, previous and concurrent infections, antibiotic and immunosuppressive therapy, urinalysis results, urinary tract instrumentation, and antifungal therapy were collected. Regarding the ethical considerations, the study was conducted on anonymous biological samples. Moreover, this research did not involve any personal data that could directly or indirectly identify a specific individual. Therefore, there was no need for the consent statement of the hospital or university ethics committee. 

The urine was treated in accordance with the recommendations of the Medical Microbiology Reference System ( 6, 7). Urinary cytology was performed on the Sysmex UF-1000i urinary cytology automated system, facilitating the quantification of leucocytes (significant threshold≥10^4^ /mL) and yeasts. In addition, microscopic examination was systematically carried out on the samples (10^3^/mL) to confirm the result and detect the presence of buddings and pseudohyphae ( 8). The culture was carried out on chromogenic medium Brillance^TM^ UTI agar (Oxoid) ( 9). If yeasts were detected in cytological examination, the cultures were extended for an additional 2-5 days to reveal the yeasts ( 10). 

**Figure 1:**
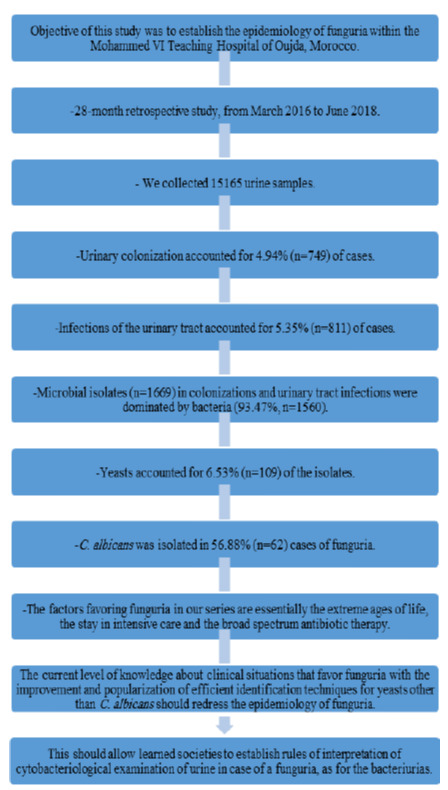
Flow diagram of the study design process and results

The triad of culture/leukocyturia/urinary tract infection (UTI) symptoms distinguished the following situations: sterile culture (absence of UTI, inflammation of the urinary tract, or antifungal treatment prior to urine collection), polymicrobial culture with three or more germs (contamination of the urine during collection), urinary colonization (asymptomatic funguria and bacteriuria), UTI (symptomatic funguria and bacteriuria) ( 6, 7). All symptomatic and asymptomatic funguria cases were confirmed based on two CBEUs. Furthermore, the isolated yeasts were identified using the BD Phoenix^TM^ Yeast ID (Becton Dickinson) biochemical galleries on the BD Phoenix^TM^ 100 (Becton Dickinson) automated system. No other identification technique was used (e.g., matrix-assisted laser desorption ionization time-of-flight mass spectrometry systems or DNA sequencing) (Figure 1). 

## Results

During the study period, a total of 15,165 urine samples were collected. The collection of mid-stream urine samples was by far the most frequently adopted method of sampling (n=12,842, 84.68%). Cultures were sterile and polymicrobial in 57.84% (n=8,771) and 31.88% (n=4834) of the cases, respectively. Urinary colonization accounted for 4.94% (n=749) of case, and UTI represented 5.35% (n=811) of the cases. Microbial isolates (n=1669) from colonization and UTIs were dominated by bacteria (n=1,560, 93.47%). 

The yeasts accounted for 6.53% (n=109) of the isolates. Table 1 shows the distribution of the yeasts based on genus and species. The most frequently isolated yeasts were *C. albicans *(n=62, 56.88%), *C. glabrata* (n=18, 16.51%), and *C. tropicalis *(n=12, 11.01%). Table 2 presents the distribution of the species isolated from funguria cases according to the recorded risk factors. This distribution was indicative of the following points: 

- The distribution of the yeasts was almost equal in all age groups. However, *Candida glabrata *particularly affected adults, mainly the elderly, more than children. 

- The distribution of the yeasts was almost equal in both genders. Nonetheless, *Candida glabrata *particularly affected females more than males. 

- Almost 43.12% (n=47) of the yeasts were isolated from the patients admitted to intensive care units. 

- Approximately one per five patients (n=21, 19.27%) had a urinary catheter. 

- More than half (n=56, 51.38%) of the patients were on antibiotic treatment. 

- Funguria was health care associated in a quarter of the cases (n=27, 24.77%). Particularly for *C. albicans*, the figure rose to one-third (n=20, 31.26%) of the cases in this regard. 

- Funguria occurred in immunocompromised patients in one quarter (n=27, 24.77%) of the cases (Table 2). 

**Table 1:** Distribution of 109 yeasts isolated from funguria cases

Genus	Species (n, %)	Funguria in UTIs		Funguria in colonization	
		n	%	n	%
*Candida*	albicans (62, 56.88%)	41	61.19	21	50.00
	*glabrata* (18, 16.51%)	11	16.42	7	16.67
	*tropicalis* (12, 11.01%)	5	7.46	7	16.67
	*parapsilosis* (7, 6.42%)	5	7.46	2	4.76
	*lusitaniae* (3, 2.75%)	1	1.49	2	4.76
	*krusei* (2, 1.83%)	2	2.99	0	0.00
	*kefyr* (1, 0.92%)	0	0.00	1	2.38
*Saccharomyces*	*cerevisiae* (4, 3.67%)	2	2.99	2	4.76

**Table 2:** Distribution of 109 yeast isolated from funguria cases according to the recorded risk factors

Fungal species (n)	Age groups			Gender		Departments	Presence of urinary catheter					Antibiotic therapy	Health care-associated infection/ colonization	Immunodepression
	Children	Young adults	Elderly adults	Female	Male	Intensive care units	Medical	Surgical	Emergency	Outpatient	No/ Yes	No/ Yes	No/ Yes	No/ Yes
*C. albicans *(62)	25	17	20	29	33	34	12	3	9	4	48/14	31/ 31	42/ 20	45/ 17
*C. glabrata* (18)	0	8	10	15	3	4	2	5	4	3	17/ 1	5/ 13	16/ 2	13/ 5
*C. tropicalis *(12)	2	8	2	5	7	6	2	0	2	2	10/ 2	6/ 6	10/ 2	11/ 1
*C. parapsilosis* (7)	3	2	2	3	4	0	3	1	3	0	5/ 2	4/ 3	6/ 1	5/ 2
*C. lusitaniae* (3)	2	0	1	1	2	2	0	1	0	0	3/ 0	2/ 1	3/ 0	3/ 0
*C. krusei* (2)	0	1	1	1	1	0	0	0	0	2	1/ 1	1/ 1	2/ 0	2/ 0
*C. kefyr* (1)	0	1	0	0	1	0	0	0	0	1	1/ 0	1/ 0	1/ 0	1/ 0
*S. cerevisiae* (4)	0	1	3	2	2	1	3	0	0	0	3/ 1	3/ 1	2/ 2	2/ 2
*Total* (n)	32	38	39	56	53	47	22	10	18	12	88/ 21	53/ 56	82/ 27	82/ 27

Children: ≤ 15 years old, young adults: 16-65 years old, elderly adults: ≥66 years old

## Discussion

The microbiological diagnosis of UTIs is established based on the CBEU. The high frequency of UTIs has made this method the most prescribed analysis in a medical microbiology laboratory ( 8). This approach is apparently simple; however, its interpretation is frequently complicated in case of the contamination of the urine with intestinal or vaginal microorganisms ( 1, 2, 5, 11). In our laboratory, this condition affected one-third of the examined urine samples. The explanation for this high rate would be the fact that the microbiological diagnosis of UTIs in our laboratory is made using a mid-stream urine sample in the vast majority of cases. This method of sampling is left to the patients themselves. They are unaware of the purpose of the CBEU, and the risk of contamination and all consequences (diagnostic delay or loss of time and money). 

In addition, while a large majority of professionals are aware of this issue, many of them do not spend enough time to explain to patients how to perform sampling, owing to their high workload. To improve the quality of the urine samples, a sampling manual was prepared and distributed to all departments in accordance with the requirements of ISO 15189, while assuming that the microbiologist is directly responsible for the entire preanalytical phase ( 11 ). This pamphlet describes all the steps of urine collection that the patient must follow to ensure the optimal quality of the collected sample. 

The second problem with CBEU interpretation is the lack of clinical information on the prescription sheet. This problem was solved by means of a mandatory electronic survey the prescriber must fill at the time of prescription in the hospital information system. This clinical information is decisive for the interpretation of CBEU ( 1). It allowed us to distinguish urinary colonization and UTIs in patients with funguria. Unnecessary antifungal treatment was spared in about 40% of patients with funguria. Funguria is only treated when it is a real UTI or urinary colonization requiring decolonization in very particular situations (pregnancy or invasive procedure programmed in urology) to prevent UTI ( 5, 12). 

The third problem related to CBEU is specific to funguria. Bacteriuria benefits from consensus recommendations established for each clinical situation suggesting significant cut-off points for each group of uropathogenic bacteria. On the contrary, this is not yet the case for the yeasts due to the rarity of therapeutic trials on this subject ( 2- 4, 13). Although some scientists adopt certain cut-offs, such as 10^4^ or 10^5^ CFU/mL ( 1- 4, 13- 18), the vast majority of them agree that only the isolation of yeasts in two successive urine samples would confirm funguria ( 2, 19). This procedure is adopted in our laboratory. The existence of a significant leukocyturia level (≥10^4^/mL) may be useful when the patient does not have a urinary catheter ( 2, 4). Similarly, the detection of buddings and pseudohyphae on microscopic examination could be a sign of yeast pathogenicity and support the diagnosis of UTI ( 2, 4). 

The distribution of the isolated yeasts showed a clear predominance of *C. albicans* (Table 1). This species remains most frequently involved yeast; however, its status has changed from a near-monopoly to a small majority ( 2- 5). As in our series, all non-*C. albicans *species now represent just under 50% of funguria ( 2- 5). The distribution of the yeasts in our series is similar to that published in the literature ( 13, 17, 18). Based on the evidence, *C. albicans *accounts for 50-70% of all *Candida* urinary isolates. *Candida glabrata*, present in approximately 10-35% of the isolates, is the second most common species, while *C. tropicalis *(10% to 35%) and C. parapsilosis (1-7%) are the less common species. However, other species are rarely isolated (1-2%) ( 13, 17, 18). 

Certain types of patients, including the elderly, have a higher risk of developing *C. glabrata* UTI more than *C. albicans *UTI ( 16- 18, 20). This is relatively in line with our findings. Risk factors for funguria are similar in most of these studies and include ICU setting, increased age, female gender, antibiotic use, urinary drainage devices, prior surgical procedures, and diabetes mellitus ( 13, 16- 18, 20). In our series, we found some of these risk factors but at varying degrees, compared to those reported in the literature. These discrepancies are likely to be due to the differences in the design of the studies. 

Previously, the identification of the yeasts was routinely performed only to distinguish *C. albicans *from "non-*albicans Candida*" using filamentation on serum ( 1, 18). This was partly the cause of the imprecise epidemiology of funguria and the lack of consensus on the subject. With the emergence of the yeasts naturally resistant to certain antifungal agents, it was necessary to specify their epidemiology using high-performance systems ( 1). The identification system used in our center allowed us to accurately identify yeasts other than *C. albicans*. This system has three main advantages: 

-It allows for better following the perpetual changes in the epidemiology of funguria. Changes explained in part by the multiplication of risk factors (i.e., urinary catheter use, diabetes, old age, female gender, malignant hematopathy, or immunosu-ppression) allow the opportunistic yeasts to express their pathogenicity ( 2- 5, 12). 

-It avoids a misleading diagnosis made based on less efficient tests (filamentation on serum, chromogenic media). For example, Saccharomyces cerevisiae has the same appearance as *C. krusei* on some chromogenic culture media ( 14, 21). 

-It allows to predict the susceptibility of the yeasts to azoles, thereby avoiding their utilization and the selection of non-susceptible (*C. krusei*) or less susceptible (*C. glabrata*) species ( 2), especially when the study of susceptibility to antifungals is not possible, as in our situation. 

Problem with biochemical identification systems, such as the one we use, is the difficulty to identify certain yeasts, such as *Candida auris* (an emergent, opportunistic, fatal, multi-resistant, and care-associated yeast), which are confused with other species of *Candida* species. Therefore, when facing with a clinical, epidemiological, and therapeutic context evoking this yeast, the use of more advanced identification techniques is essential (MALDI-TOF MS systems or DNA sequencing) ( 22). 

## Conclusion

In the face of funguria, the distinction between infection, colonization, and simple contamination is not always easy. The lack of consensus on significant funguria levels is the main cause of this issue. The enhancement of the current level of knowledge on the clinical situations that lead to funguria, as well as the improvement and popularization of high-performance techniques for the identification of yeasts other than C. albicans, should redress the epidemiology of funguria. This should allow societies to establish the rules of CBEU interpretation in the event of funguria, such as bacteriuria. 

##  Authors’ contribution

All authors have made substantial contributions to the conception and design of the study, as well as data acquisition, analysis, and interpretation. Additionally, equal contributions have been made by all authors in drafting the article and revising it critically for important intellectual content, and the final approval of the version to be submitted.

## Conflict of Interest:

None.

## Financial disclosure

There are no financial conflicts of interest to disclose.
